# Extracellular *Streptomyces lividans* vesicles: composition, biogenesis and antimicrobial activity

**DOI:** 10.1111/1751-7915.12274

**Published:** 2015-04-07

**Authors:** Hildgund Schrempf, Philipp Merling

**Affiliations:** FB Biology/Chemistry, Applied Genetics of Microorganisms, University OsnabrückBarbarastr. 13, D-49069, Osnabrück, Germany

## Abstract

We selected *Streptomyces lividans* to elucidate firstly the biogenesis and antimicrobial activities of extracellular vesicles that a filamentous and highly differentiated Gram-positive bacterium produces. Vesicle types range in diameter from 110 to 230 nm and 20 to 60 nm, respectively; they assemble to clusters, and contain lipids and phospholipids allowing their *in situ* imaging by specific fluorescent dyes. The presence of the identified secondary metabolite undecylprodigiosin provokes red fluorescence of a portion of the heterogeneous vesicle populations facilitating *in vivo* monitoring. Protuberances containing vesicles generate at tips, and alongside of substrate hyphae, and enumerate during late vegetative growth to droplet-like exudates. Owing to *in situ* imaging in the presence and absence of a green fluorescent vancomycin derivative, we conclude that protuberances comprising vesicles arise at sites with enhanced levels of peptidoglycan subunits [pentapeptide of lipid II (C55)-linked disaccharides], and reduced levels of polymerized and cross-linked peptidoglycan within hyphae. These sites correlate with enhanced levels of anionic phospholipids and lipids. Vesicles provoke pronounced damages of *Aspergillus proliferans*, *Verticillium dahliae* and induced clumping and distortion of *Escherichia coli*. These harmful effects are likely attributable to the action of the identified vesicular compounds including different enzyme types, components of signal transduction cascades and undecylprodigiosin. Based on our pioneering findings, we highlight novel clues with environmental implications and application potential.

## Introduction

*Streptomyces* species are highly differentiated Gram-positive bacteria that are highly abundant in soils. As they have a large repertoire of extracellular enzymes, they play an important ecological role in the turnover of organic molecules, including complex mixtures of high-molecular weight compounds. In addition, streptomycetes secrete proteins including those with high affinity for specific polysaccharides that participate to target other organisms including fungi that are the focus of several studies (Siemieniewicz and Schrempf, 2007; Siemieniewicz *et al*., 2007; Lamp *et al*., 2013). Like other bacteria (Filloux, 2010), streptomycetes secrete diverse proteins via the Sec pathway or via the Tat machinery. In addition, only a type VII system was studied in a *Streptomyces* strain (Akpe San Roman *et al*., 2010; reviews: Yuan *et al*., 2010; Chater *et al*., 2010).

Streptomycetes produce an impressive repertoire of chemically diverse compounds, so-called secondary metabolites that inhibit many cellular reactions including DNA replication, transcription and protein synthesis in many organisms, as well as cell wall synthesis of bacteria and certain fungi (reviews: Kutzner, 1981; Schrempf, 2007; Chater *et al*., 2010).

Depending on their chemical features, low molecular weight secondary metabolites are either associated to the *Streptomyces* hyphae or present within the culture filtrate. The uptake of these compounds occurs via members of ABC transporters or, respectively, of the major facilitator superfamily (review: Martín *et al*., 2005).

Previously, we found for the first time that a *Streptomyces* strain – i.e. *Streptomyces coelicolor* M110, a derivative of *S. coelicolor* A3(2) – produces a dense array of many vesicles that accumulate in droplet-like exudates on sporulating lawns. As determined by transmission electron microscopy (TEM) and cryo-electron microscopy, the vesicle diameters vary between 80 and 400 nm. Attributed to additional studies, we concluded that the vesicles vary in their structure and macromolecular composition, both membrane-associated and soluble material. Using biochemical tools, we revealed that the assemblies of vesicles contained the polyketide actinorhodin, and high concentrations of distinct proteins. These were deduced to be necessary for the acquisition of inorganic as well as organic phosphate, iron ions, of certain carbon sources, energy metabolism and redox balance, defence against oxidants and tellurites, the tailoring of actinorhodin, folding and assembly of proteins, establishment of turgor, and different signalling cascades (Schrempf *et al*., 2011).

Streptomycetes build tangled hyphae networks, so-called pellets. In contrast to *S. coelicolor* M110, *Streptomyces lividans* builds less tangled hyphae networks (Koebsch *et al*., 2009) allowing to follow the growth of individual filaments more distinctly. In addition, *S. coelicolor* M110 produces high levels of an extracellular agarase (Chater *et al*., 2010) that leads to local sinking of agar containing substrate hyphae that are subsequently more difficult to be analysed individually. Therefore, we selected in frame of this report, *S. lividans* as a more suitable model strain to investigate the *in vivo* generation of extracellular vesicles by fluorescence microscopy without fixation or antibody treatment. We succeeded to elucidate firstly principles concerning the biogenesis of *Streptomyces* vesicles, their interaction to large assemblies and their role in killing some other microbes.

## Results

### *Streptomyces lividans* exudates contain densely packed vesicles of different types

**Fig 1:**
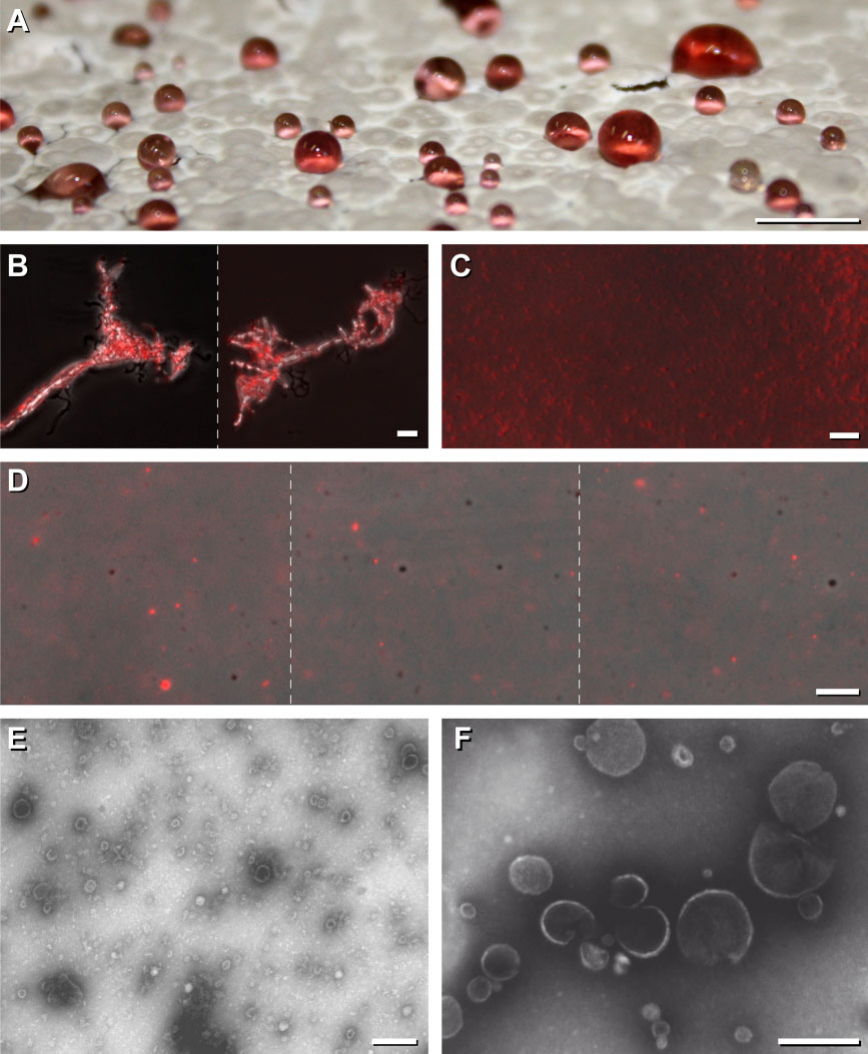
Macroscopic and microscopic inspection of *S. lividans* exudates.A. *Streptomyces lividans* was grown on agar-containing medium for 7 days. Using a macro lens, a photo was taken from the top of the *S. lividans* lawn containing red droplets. Bar: 5 mm.B–D. Aliquots from the droplets (see, A) were inspected microscopically by phase contrast under visual light, and for the presence of endogenous red fluorescence. Then, pictures were merged (B–D). Regions from undiluted (B, C) and diluted (D) samples are presented. Bars: 5 μm.E, F. Aliquots of the sample (corresponding to Fig. 1C) were prepared on grids (see, *Experimental procedures*), and inspected by TEM at two different magnifications (E and F). Bars: 200 nm.

In the course of cultivating *S. lividans* for 6–7 days, droplets arose on top of sporulated areas on agar plates (see *Experimental procedures*). The volume of visible droplets usually contained 5–40 μl; their colour was reddish (Fig. 1A). Following extraction, we identified ions m/z (394.4 M + H)^+^ by LC/MS that are characteristic for the prodiginine-type undecylprodigiosin (Meschke *et al*., 2012). Microscopical investigations revealed that exudates contained condensed areas that exhibited an intensive red fluorescence (Fig. 1B) that consist of roundish particles (Fig. 1C). Individual particles exhibited either red or no fluorescence (Fig. 1D). Based on the above findings together with the previously identified spectral features of prodiginine types (Meschke *et al*., 2012), we conclude that the red fluorescent particles contained undecylprodigiosin. These had a diameter of ∼250 nm or smaller, and therefore, being at or below the detection limit by light microscopy. Sometimes larger elements, possibly resulting from clustering of smaller ones, were present.

Detailed investigations by TEM revealed that the droplets (Fig. 1A) contain high numbers (∼10^11^ μl^−1^) of larger and smaller roundish particles (Fig. 1E). Larger particles had a diameter ranging from 110 to 230 nm, and frequently, they had a lighter appearing ring that is reminiscent to membranes (Fig. 1F). The particles appeared sometimes broken; therefore, we cannot exclude that the spectrum of smaller subtypes (20–60 nm diameters) with different shapes can originate from their interior during the preparation procedure. In addition, larger clusters were present (Fig. 1E and F).

**Fig 2:**
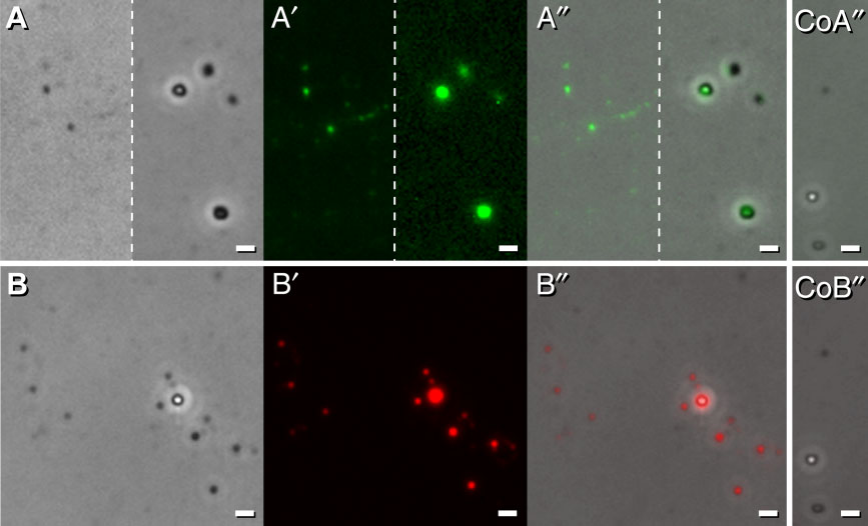
Analyses of droplet samples following staining by NAO and Nile red, or by transmission electron microscopy (TEM).A-CoA″. A sample of a droplet (see Fig. 1A) was treated with the dye NAO (10-N-Nonyl Acridine Orange), and inspected microscopically under visual light (A), for the presence of green fluorescence (A′), or analysed after merging (A″). The control was done without staining, and is presented as merged picture (CoA″). Bars 2.5 μm.B-CoB″. A sample (corresponding to Fig. 1D) was treated with Nile red, and it was analysed microscopically under visual light (B), for the presence of red fluorescence (B′), and after merging (B″). The endogenous undecylprodigiosin derived fluorescence was considerably fainter than that one obtained after Nile red staining, and hence, undetectable in the unstained reference (control CoB″) under the set photographical conditions that were used to detect specifically Nil red derived fluorescence. Bars 2.5 μm.

Phospholipids are typically present within membranes of bacteria including those of streptomycetes (Hoischen *et al*., 1997). Recent investigations of *Escherichia coli* under physiological conditions revealed that the 10-N-Nonyl Acridine Orange (NAO) binds to structural different anionic phospholipids, but is insensitive to their structure. The identified lack of specificity is attributed to the fact that phosphate group(s) from these molecules mediate a 1:1 charge interaction with the fluorophore leading to a considerable *Stokes* shift of NAO fluorescence. The green shift was most pronounced, whereas the red shift was considerably lower, and did not correspond to one specific phospholipid type as deduced from previous *in vitro* studies (Oliver *et al*., 2014). Therefore, we applied NAO to the particles (corresponding to those within Fig. 1), and inspected them under visual light by phase contrast (Fig. 2A), and under fluorescent light for the *Stokes* shift. The particles had acquired an intensive green fluorescence (Fig. 2A′, and merged 2A″) whereas those within the control (Fig. 2CoA″) lacked this feature. Additionally, we detected a weaker NAO dependent red shift (not shown). The results suggested that the identified particles contain anionic phospholipids lipids.

Nile red is non-florescent in water, and exhibits red fluorescence in a lipid-rich environment (Kumagai *et al*., 2005). In order to test for the presence of lipophilic compounds, we treated vesicles (corresponding to Fig. 1A) with Nile red, and analysed them by visual light (Fig. 2B) and for the presence of red fluorescence (Fig. 2B′), and after merging of the resulting pictures (Fig. 2B″). Nile red induced strong fluorescence (Fig. 2B′), which was about six times higher than to that one recorded for the endogenous undecylprodigiosin. Considering these differences small gain values, we succeeded to score for the specific intensive fluorescence following treatment by Nile red (Fig. 2B′ and B″) in comparison to the unstained control (Fig. 2CoB″). Additional experiments revealed that the particles gained specific red fluorescence following treatment with the dye FM4-64 that was weaker (data not shown) than that one obtained after application of Nile red. FM4-64 belongs to a class of amphiphilic styryl dyes that fluoresce in a hydrophobic environment, and hence, has a pronounced membrane selectively (Bolte *et al*., 2004). Taking together, the data revealed that the vesicles contain lipophilic compound(s).

### Clusters of vesicle-like particles emerge from tips and alongside of substrate hyphae

**Fig 3:**
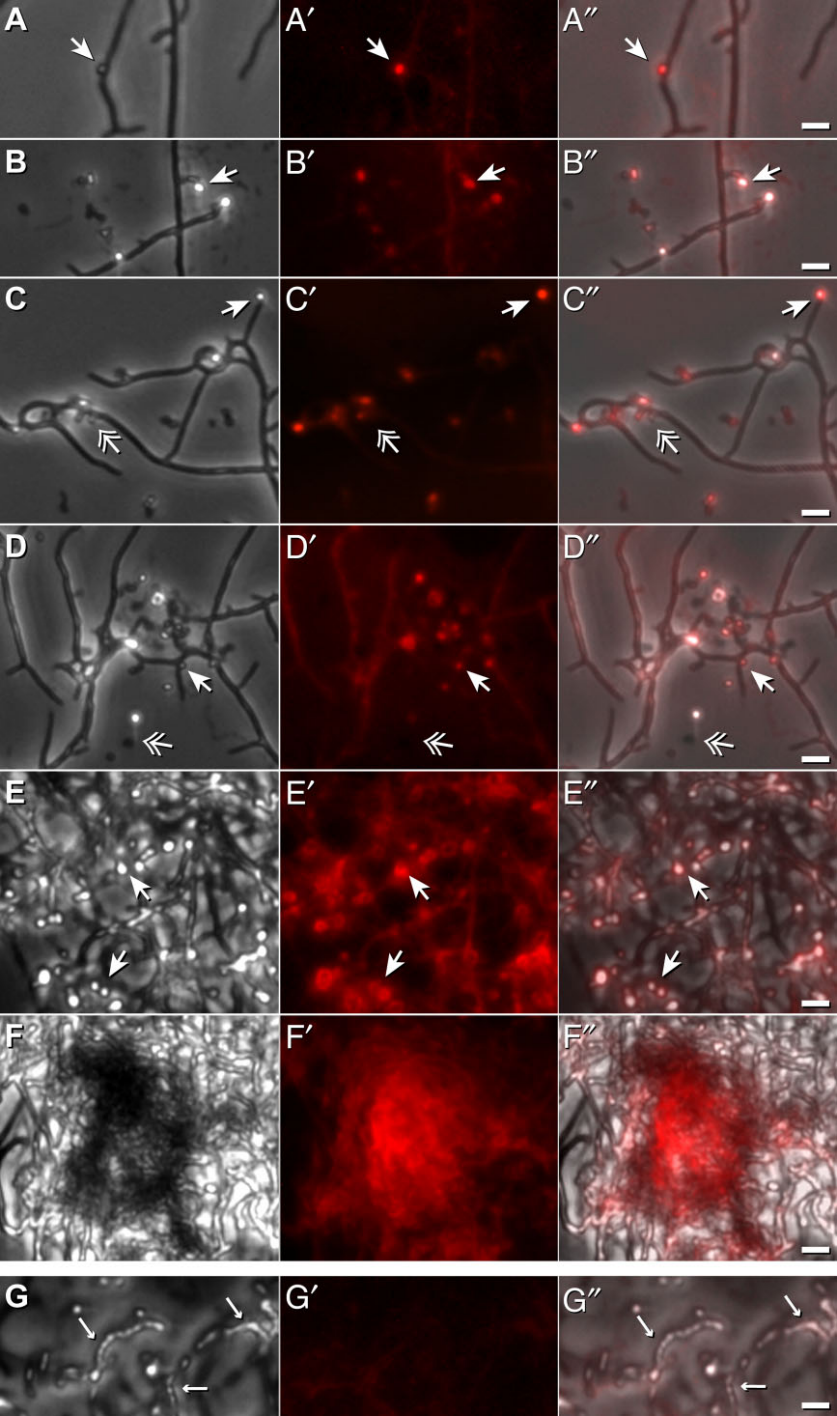
Formation of vesicle-like structures during development of *S. lividans*.A–F″. Spores were seeded onto agar plates, and incubated as described under *Experimental procedures*. Samples were inspected by phase contrast after 2.7 (A), after 4–5 (B–E) and after 6 (F) days within areas containing a few (A, B), moderate (C, D) or high numbers (E, F) of substrate hyphae. In addition, the samples were scored for endogenously derived red fluorescence (A′–F′), and merged with A–F to result in A″–F″. Different types of arrows mark particles that have a red fluorescence (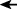), or none (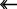). Bars: 2.5 μm.G. Aerial hyphae (←) lack intensive red fluorescence (G′ and G″). Bars: 2.5 μm.

In order to get novel insights concerning the biogenesis of the vesicles, we inspected *S. lividans* germinating spores during their development to substrate and aerial hyphae. After growth for about 60 h, a few small roundish bulges (∼500 up to ∼1000 nm in diameter) were detectable under visual light by phase-contrast (Fig. 3A) and by their red fluorescence (Fig. 3A′ and merged A″) at a few substrate hyphae. Their numbers increased by extending the cultivation time for 4–5 days. They were most easily visible within areas containing small numbers of hyphae where they appeared alongside them as well as at their tips, or at starting points for branches (Fig. 3B, B′, B″, C, C′ and C″). The fluorescent and roundish protuberances were more easily detectable than co-evolving non-fluorescent ones; both types appeared sometimes as released forms (Fig. 3C, C′, C″, D, D′ and D″). The number of fluorescent clusters rose within areas of higher hyphae density (Fig. 3D, D′ and D″), and increased considerably within very tangled hyphae networks (Fig. 3E, E′ and E″). Large patches of intense red fluorescent conglomerates arose after 6 days of growth (Fig. 3F, F′ and F″) that developed to the exudates comprising the analysed vesicles (Figs 1 and 2). Noticeably, the co-emerging aerial hyphae with spore chains were devoid of red fluorescence (Fig. 3G, G′ and G″).

### Lipid- and phospholipid-rich sites at the substrate hyphae correlate with clusters of vesicle-like particles

**Fig 4:**
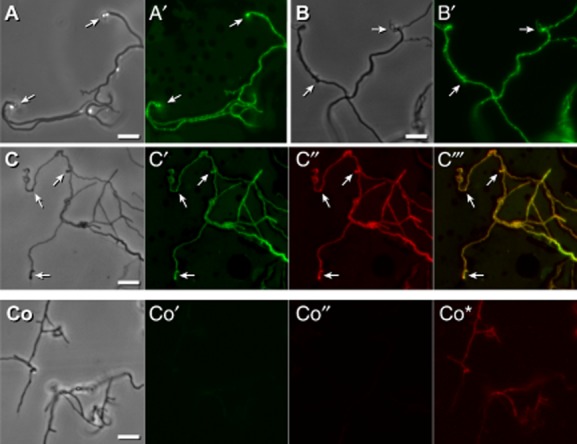
Detection of nascent vesicles using lipid-specific dyes. *Streptomyces lividans* was grown (see Fig. 3) for 4 days.A, B′. Two samples of hyphae were treated with NAO, and inspected microscopically under visual light (A, and B) as well as for the presence of green fluorescence (A′ and B′). Pronounced bulged structures are marked (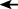).C,C‴. Hyphae were treated with NAO and Nile red, and inspected microscopically under visual light (C), for the presence of NAO derived green fluorescence (C′), for Nile red derived strong red fluorescence (C″), and following merging (C‴) of both types of fluorescence. Pronounced protuberance-structures are marked (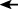).Co–Co*. Untreated control hyphae (Co) lacked green fluorescence (Co′). If analysed under the same gain value that corresponded to C″, the hyphae were not red fluorescent (Co″) showing that the detection of Nile-red dependent staining (C″) was specific. However, if four times higher gain value were applied, some endogenous red fluorescence (Co*) was detectable. Bars: 5 μm.

Substrate hyphae growing (Fig. 4A and B) for 4 days were treated with NAO. Highly pronounced green florescence occurred at the tips or at sites at the hyphae that were detectable by phase contrast as bulges (Fig. 4A, A′, B and B′). Additionally, regularly spaced regions appeared within the hyphae whose green fluorescence was more elevated than the overall one.

Co-treatment with NAO and Nile red revealed that both dyes co-located as pronounced red and green fluorescent sites at substrate hyphae where the bulges (∼500 up to 1000 nm in diameter) arose (Fig. 4C–C″). These locations corresponded to those of the endogenously derived undecylprodigiosin-dependent fluorescence (Fig. 3A″–D″). This was about six times fainter (Fig. 4Co*) than that one obtained after Nile red staining, and it was undetectable if small gain values were applied for recording (Fig. 4Co″). Considering the dimensions of individual vesicles (Figs 1 and 2), we conclude that bulges contain vesicle assemblies. The control hyphae lacked green florescence (Fig. 4Co′).

### Vesicle-like particles extrude at sites with enhanced concentrations of peptidoglycan subunits

**Fig 5:**
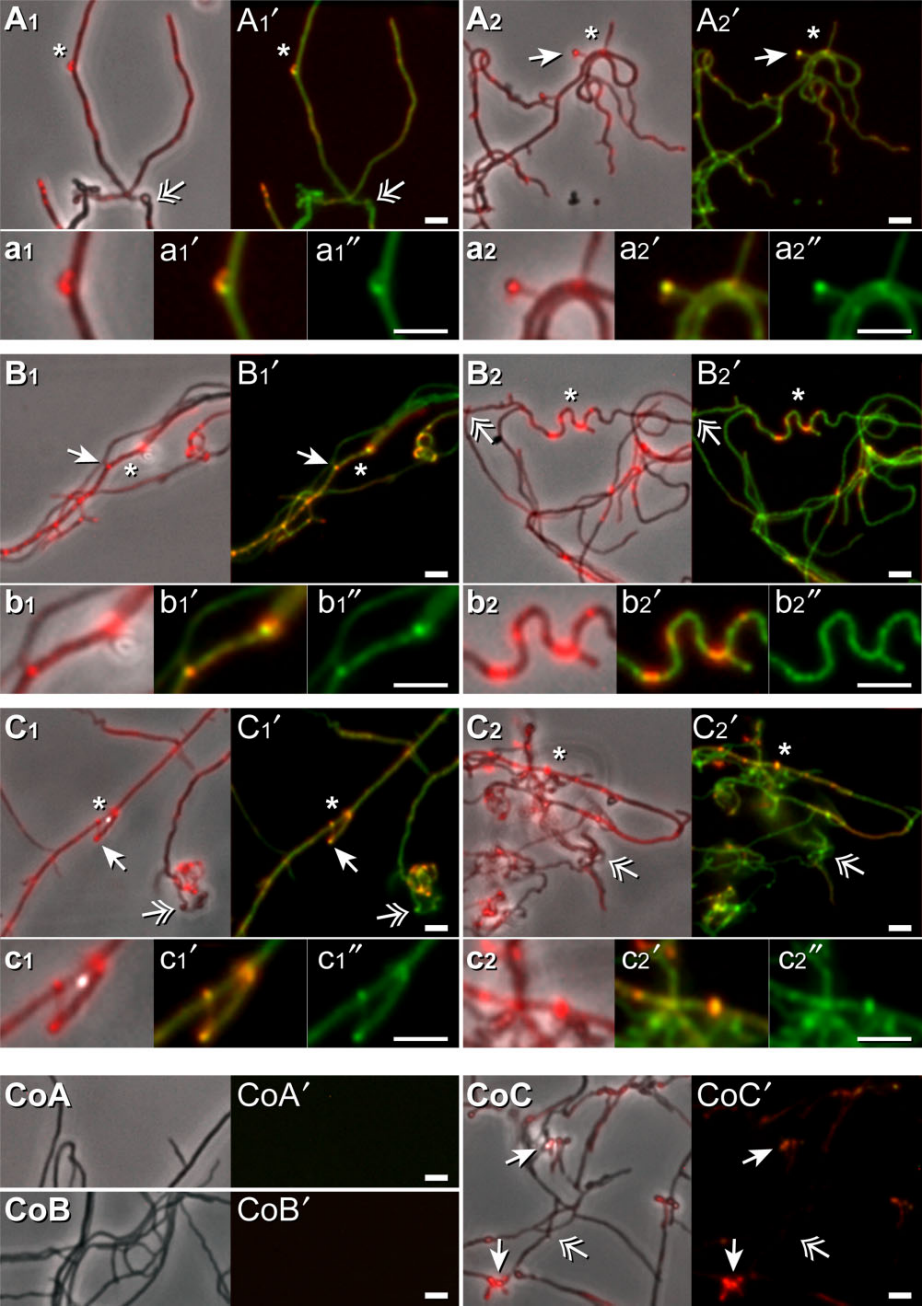
*In situ* effect of vancomycin.A–C2. Spores were seeded on agar plates as described under *Experimental procedures*, and incubated for different time periods: 20 h (A1-a2″), 42 h (B1-b2″), and 5 days (C1-c2″). Samples were treated with Van-FL, analysed under visual light by phase contrast, and scored for red fluorescence (due to endogenous undecylprodigiosin), merged (A1, A2, B1, B2, C1 and C2) and presented as magnified subareas (a1, a2, b1, b2, c1 and c2). In addition, the Van-FL-derived green fluorescence of the same samples was recorded, and merged with their red florescence (endogenous undecylprodigiosin, see above); this procedure resulted in the pictures A1′, A2′, B1′, B2′, C1′ and C2′ with corresponding magnified regions (a1′, a2′, b1′, b2, c1′, and c2′). Additionally, the magnified areas showing only Van-FL derived fluorescence are shown (a1″, a1″, b1″, b2″, c1″ and c2″). Positions containing droplet-like structures with a fluorescence (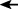) or none (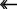) are marked. Fluorescence sites (*) that were additionally enlarged are marked. Bars: 2.5 μm.CoA-CoC′. Control (Co) samples corresponding to A, B, and C, which were not treated with Van-FL were analysed under visual light by phase contrast (CoA, CoB and CoC), and inspected for red and green fluorescence light. The resulting pictures were merged. (CoA′, CoB′ and CoC′). Bars: 2.5 μm.

Attributed to the results presented in the previous chapter, we liked to explore if the extrusion of the vesicle-like particles can occur at not cross-linked sites within the peptidoglycan that arise during the nascent state of biogenesis, or at late growth stages due to the action of hydrolytic enzymes. The antibiotic vancomycin tagged with the green fluorescent dye BODIPY FL (Van-FL) is a suitable probe for local sites of un-polymerized peptidoglycan (Daniel and Errington, 2003); hence, we used it for the designed *in vivo* monitoring. Young *S. lividans* hyphae that developed from seeded spores after 20 h interacted with Van-FL along the substrate hyphae. However, highly intense green Van-FL fluorescence arose at a few sites with kinked or bent appearances, and sometimes at hyphae tips. The positions of these sites correlated with roundish protuberances or bulges (∼500 to ∼1000 nm). The non-fluorescent ones were more difficult to detect compared with those exhibiting undecylprodigiosin-derived red fluorescence (Fig. 5A1, A1′, and a1, a1′, and A2, A2′, and a2, a2′). Extension of the cultivation time to 42 h resulted in an increase of sites positioned in zigzag or wave-like areas that had simultaneously both fluorescence types (Fig. 5B1, B1′, and b1, b1′, and B2, B2′, and b2, b2′). The control hyphae grown for corresponding time periods had fewer kinks, and lacked endogenous red and green fluorescence (Fig. 5 CoA, CoA′, and CoB, CoB′).

After 5 days of cultivation, the hyphae built a dense network, and therefore, individual ones within less condensed areas were chosen for inspection. Following the addition of Van-FL, the hyphae appeared either moderately or highly bent and coiled; the numbers of red fluorescent bulges increased in addition to some non-fluorescent ones, but both types stained with Van-FL (Fig. 5 C1, C1′, c1, c1, and C2, C2′, c2, c2′). In contrast, the untreated control hyphae were less bent, produced fewer numbers of sized red fluorescent protuberances (Fig. 5CoC and CoC′) and their overall shape corresponded to those presented under Fig. 3D, D′ D″ (see, a previous chapter).

### Vesicle containing exudates contain high concentrations of different proteins

Vesicle-containing droplets (Fig. 1A and C) contained 1–2 μg protein μl^−1^. Following electrophoresis, distinct protein-containing bands were detectable. Following in-gel digestion with trypsin, we analysed the resulting peptides by LC/MS (Aebershold and Mann, 2003). The population of subsequently deduced proteins contained dominantly different types of predicted enzymes, and a few others (Table 1).

**Table 1:** List of identified proteins

No. STRLI	MW (kDa)	Deduced function	Remarks
WP_016326180	115.377	Peptidase S41 family	No
D6EFC0	75.730	Phospholipase C-type	T
D6EU9	74.106	Peptidase S1 type	S
D6EK57	64.407	Nucleotidase/apyrase	S
D6EJ35	59.538	Alkaline Phosphatase (PhoD-like)	T
D6E187	58.495	Substrate binding protein family 5	S
D6EKU8	55.399	Esterase FusH	S
D6EG84	55.130	Catalase (Kat E-type)	No
D6EUN8	52.193^a^	Nucleotide binding protein (cNMP)	T
D6EHM2	49.559	Pyridine-nucleotide-disulphide oxidase family	No TM
D6ENU1	43.375	Glycerophosphoryl-diester-phosphodiesterase	T
Q4A4I7	38.041	Phosphate binding protein PstS	T
D6EXX3	36.642	Binding protein (FE_B12_PBP)	T
D6ERQ9	36.245	Ketol-acid reductoisomerase	No
D6ENL9	34.936	Peptidyl-prolyl cis-trans isomerase	L
D6EXY3	34.302	Agmatinase	No
D6EFX5	20.388	Ter D-like protein	No TM

a Gained from gel only truncated (∼23 kDa).

L, predicted lipoproteins; No, no predicted signal peptide; S, predicted Sec signal peptide; T, predicted Tat signal peptide; TM, transmembrane region.

The identified peptidases belong to the family S41, and respectively to the S1 type. Several proteins that are necessary for the metabolism of phosphates, or selected compounds with phospho-group(s), include a phospholipase C-type, an alkaline phosphatase type, a glycerophoshoryl-diester-phosphodiesterase and a phosphate binding protein. We detected the esterase FusH, a catalase (KatE type), a member of the pyridine-nucleotide-disulphide oxidase family, one ketol-acid reductoisomerase, a peptidyl-propyl cis-trans-isomerase as well as an agmatinase. In addition, a nucleotide binding protein, and a binding protein of the FE-B12 group were present. Proteins contain either a predicted (Sec or Tat) signal sequence or none. Two proteins contain transmembrane regions, and one enzyme has a predicted lipid anchor. The data show that the vesicles are well equipped with different proteins (Table 1). As examples, we tested activities of peptidase (with azocasein) and alkaline phosphatase (Lamp *et al*., 2013). Both enzyme types were highly active (data not shown).

### Vesicle exudates provoke clumping of *E. coli* but not *Bacillus subtilis*

**Fig 6:**
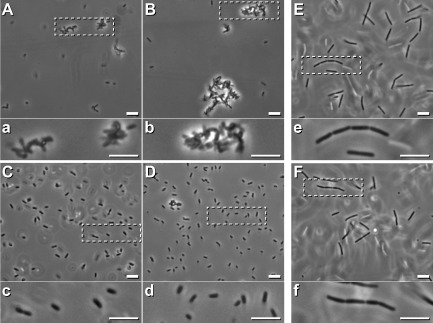
Applications of vesicle-containing droplets to *E. coli* and *B. subtilis*.A–D. An aliquot (500 μl) of logarithmically grown *E. coli* was supplemented with a sample (20 μl) of vesicle-containing droplets (see Fig. 1A, and C), incubated at 30°C, and analysed microscopically after 2 min [A and its framed enlarged (a), area], and respectively after 40 min (B, and enlarged area, b). Controls were done (C, c, and D, d) without the addition of droplet. Bars 5 μm.E, F. A portion of logarithmically grown *B. subtilis* was incubated at 30°C for 2 h with a sample of vesicle-containing droplets (E, and e), or without them as a control (F, and f), and presented in two different magnifications. Bars 5 μm:

The addition of the vesicle-containing exudates (Fig. 1A and C) to logarithmically growing *E. coli* DH5α induced within 2 min cell clumps (Fig. 6A, a) whose sizes increased during incubation up to 40 min that appeared distorted (Fig. 6B, b) in contrast to the controls (Fig. 6C, c, and D, d). The clumps and distortions persisted overnight. Treatment with LIVE/DEAD stains (see *Experimental procedures*) revealed that cells were alive, as they did not take up propidium iodine (PI) like the untreated control cells. As an additional control, we mixed an aliquot of the *E. coli* culture with vesicles and PI, and then, added toluene (5%) in order to provoke an artificial cell permeability. As a result, cells gained instantly PI-derived intensive red fluorescence that corresponded to control cells that were only treated with PI and toluene (not shown). Based on this finding, we exclude that vesicular compounds modify or trap PI. This result prompted an additional streaking on agar plates. The number of offspring's that grew overnight from the treated and untreated cells was comparable. Taken together, the results clearly demonstrated that vesicles provoked clumping and distortion of *E. coli* cells (Fig. 6A and B) while they remained viable.

In contrast, the application of exudates (Fig. 1A and C) to the Gram-positive *B. subtilis* within its logarithmic phase after short (not shown) or continuing incubation for 2 h (Fig. 6E, e, and control F, f) lacked an effect.

In contrast to the tested fungi (see next chapter), neither *B. subtilis* nor *E. coli* cells accumulated prodigiosin following incubation with vesicles.

### Vesicle exudates provoke lesions in *Aspergillus proliferans* and *Verticillium dahliae*

**Fig 7:**
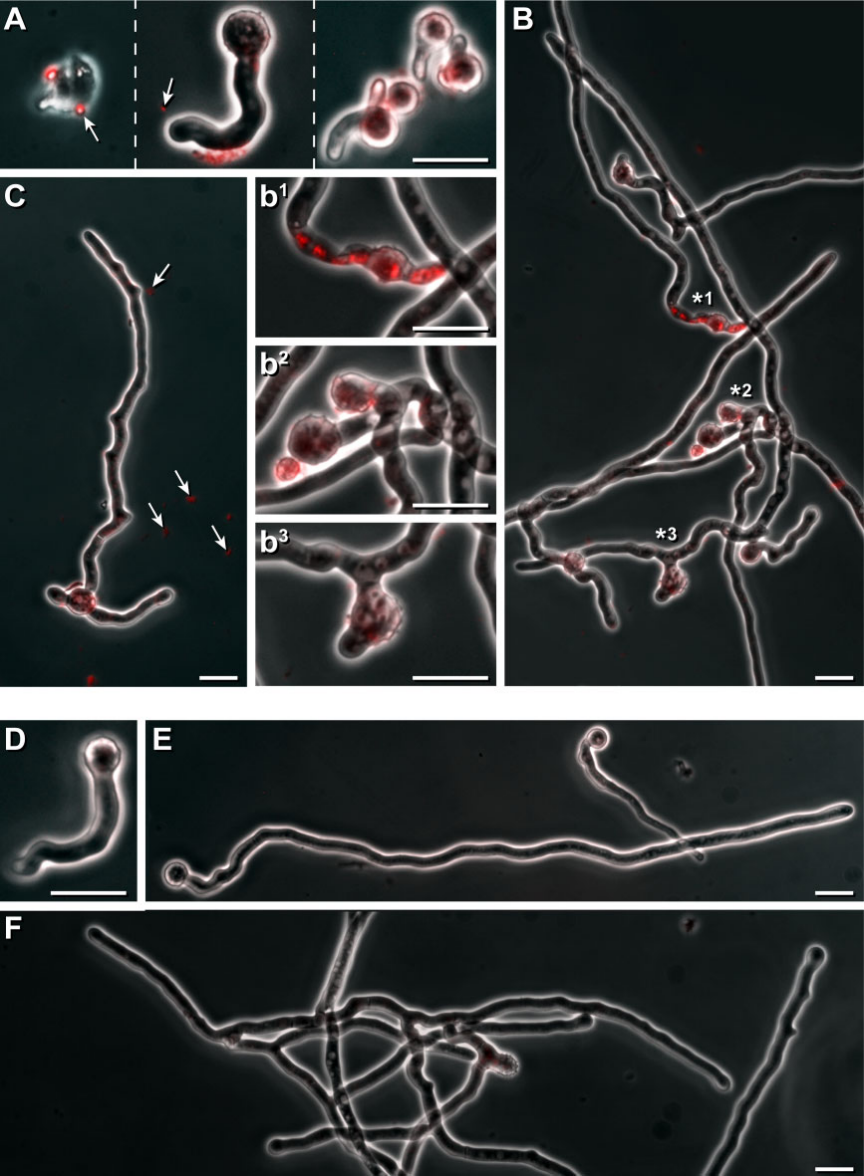
Effect of vesicle-containing droplets on *A. proliferans*.A–C. Conidia of *A. proliferans* were incubated 4 h in liquid medium. Then, an aliquot (20 μl) of vesicles containing droplets (see Fig. A and C) was added to a culture aliquot (500 μl), and incubation continued for additional 3 h incubated for 2 h at 30°C. (A) or for 8 h (B, C). All samples were inspected microscopically by phase contrast as well as for undecylprodigiosin-derived red florescence, and are shown in a merged fashion. Vesicle-like particles that are red fluorescent (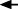) are marked. Regions from the hyphae (B) with high red intracellular fluorescence (*1, or that had developed blown up (*2), or strongly deformed (*3) areas are presented in an enlarged fashion (b^1^, b^2^ and b^3^). Bars 10 μm.D–F. Controls were without the addition of droplets, and inspected as outlined above. Bars 10 μm.

Conidia of *A. proliferans* were pre-incubated in liquid medium for 4 h. Following the addition of vesicle-containing droplets (Fig. 1A and C) for 3 h, red fluorescent particles (∼500 up to 1000 nm) were sometimes adhering to conidia that were not yet germinated (Fig. 7A, left). In addition, red florescence was detectable at conidia and germination tubes (Fig. 7A, middle and right). If the incubation time with the vesicle-containing exudates extended to 8 h, distinct red fluorescent compact round areas appeared within the hyphae (Fig. 7B, and enlarged b1). In addition, blown up areas with different shapes were present (Fig. 7B, enlarged b2 and b3). Some hyphae had small local deformations, leading to their kinky shape (Fig. 7C). The corresponding control germination tubes (Fig. 7D) as well as the hyphae were devoid of red fluorescence and the above-outlined deformations (Fig. 7E and F).

**Fig 8:**
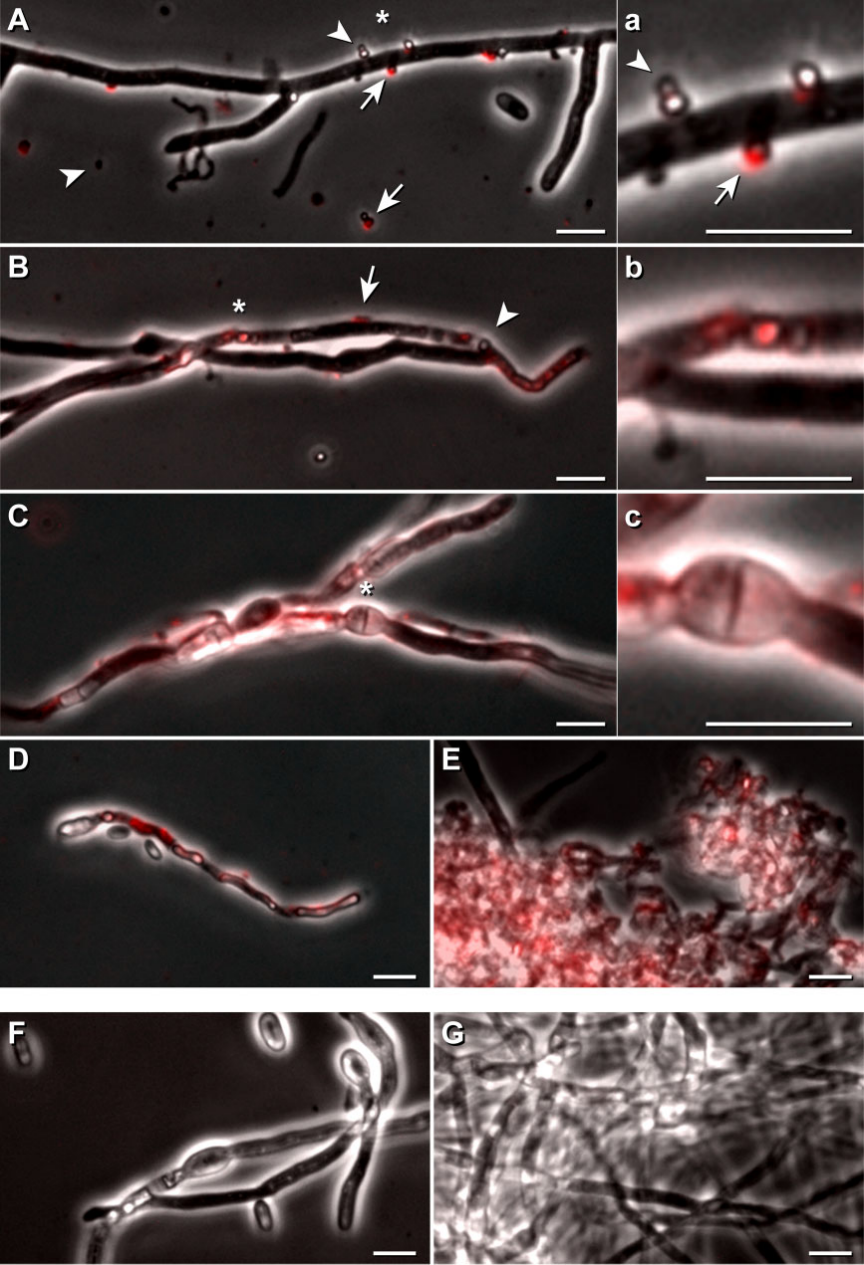
Effect of vesicle-containing droplets on *V. dahliae*.A,B. An aliquot (20 μl) of vesicle-containing droplets (see Fig. 1A and C) was added to an aliquot (500 μl) of a *V*. *dahliae* culture, and incubated for 2 h at 30°C. Samples were inspected microscopically by phase contrast, and for the presence or absence of undecylprodigiosin-derived red florescence. Subsequently, pictures were merged. Selected areas (*) from A, and B were enlarged, and are presented in a and b. Vesicles adhering to fungal hyphae had either a red (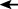) or no (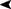) fluorescence. Bars 5 μm.C–E. Pre-grown fungal hyphae were incubated for 20 h with vesicles containing droplets, and inspected as described above. An area (*) of C is presented additionally in an enlarged fashion in c.F–G. Controls correspond to cultures C and E without the addition of droplets, and these were inspected as outlined above. Bars 5 μm.

Upon adding an aliquot of the exudates (Fig. 1A and C) for 2 h to *V. dahliae* hyphae, vesicle clusters (∼500 nm to ∼1000 nm) without or with red fluorescence were found sometimes aligned along the hyphae (Fig. 8A, a and B, b). Hyphae regions that appeared to have taken up vesicles (Fig. 8B and b) had thinned and kinked regions (Fig. 8B). The prolongation of the incubation time for 20 h resulted in red fluorescence within hyphae and arising bulges (Fig. 8C and c). Hyphae and co-emerging conidia were often short and thinned (Fig. 8D), and many bulged structures dominated (Fig. 8E). The corresponding controls were devoid of red fluorescence; the hyphae and the developing conidia had a wider diameter (Fig. 8F as control for D), and dense hyphae networks were build (Fig. 8G as control for E).

## Discussion

In frame of the presented study, we deepen the knowledge on extracellular vesicles from streptomycetes. Investigations by TEM revealed that the diameter of *S. lividans* vesicles ranges from 110 to 230 nm, and respectively, 20 to 60 nm (Fig. 1). While the generation of *S. coelicolor* M110 vesicles correlates with the dominant secretion of the non-fluorescent polyketide actinorhodin (Schrempf *et al*., 2011), *S. lividans* produces under corresponding conditions a large portion of vesicles that contain undecylprodigiosin (Fig. 1), a prodiginine type from which we had previously determined the spectrum of its red fluorescence upon uptake in cells (Meschke *et al*., 2012).

During early growth stages, *S. lividans* produces rarely small roundish, often red fluorescent sphere-like protuberances that enumerate considerably upon prolonged cultivation time, and ultimately, develop to intensely red fluorescent conglomerates (Fig. 3), and then, to droplet-like exudates (Fig. 1). These enclose masses of vesicles (∼10^11^ μl^−1^) having anionic phospholipids and neutral lipids (Figs 2 and 4). Considering the diameters of the vesicles (Fig. 1), we deduce that the size of bulges (∼500 to 1000 nm) at the hyphae comprise an assembly of a few larger or a mixed population of vesicles. Possible smaller protuberances are under the detection limit during the *in vivo* investigations by fluorescence microscopy.

Our studies revealed that the presence of the green fluorescent vancomycin derivative (Van-FL) enhanced the number of vesicle-containing bulges at the *S. lividans* substrate hyphae, and shifted their formation to earlier growth periods (Fig. 5). It is known that the glycopeptide antibiotic vancomycin specifically targets the C-terminal D-alanine-D-alanine within the precursor pentapeptide of lipid II (C55)-linked disaccharides (peptidoglycan subunit) (Hasper *et al*., 2006). This action provokes an impaired binding of proteins that are required to catalyse the correct integration and polymerization of peptidoglycan subunits. The resulting inhibition of cell wall synthesis provokes cell lysis (reviews: Lovering *et al*., 2012; Manat *et al*., 2014). In agreement with this knowledge is our finding that following the addition of vancomycin, *S. lividans* substrate hyphae have a highly curved and deformed appearance, and extrude high levels of bulges (Fig. 5) corresponding in sizes to those that arise without vancomycin at later growth stages (Fig. 3 and controls in Fig. 5). It is known that bacteria produce an array of enzymes (hydrolases or autolysins) during expansion and repair of the peptidoglycan layers of which many are present in the cell wall of Gram-positive bacteria (review: Uehara and Bernhardt, 2011). Based on this knowledge and our data, we conclude that the vesicle-containing protuberances arise at sites containing defects within the peptidoglycan layer of *Streptomyces* substrate hyphae.

Previous studies of the Gram-positive *B. subtilis* indicated changes of lipid domains (Matsumoto *et al*., 2006) within the cytoplasmic membrane of cells having a disrupted peptidoglycan (Muchová *et al*., 2011). Assuming a corresponding correlation in *Streptomyces*, it appears likely that defects in cell wall synthesis during late stages of growth, or following treatment with vancomycin (Figs 3 and 5), result in an alteration of lipid domains, and therefore, facilitate the formation of the vesicle-containing protuberances that we found to have enhanced levels of anionic phospholipids and neutral lipids (Fig. 4).

In culture filtrates from a few investigated Gram-positive bacteria (i.e. *B. subtilis*, *Staphylococcus aureus*), membrane vesicles (MVs) are present; however, the mode of their biogenesis is unknown. A range of Gram-negative bacteria produce outer membrane vesicles (OMVs); however, despite many efforts, the exact mode of their formation is not yet determined. This deficit is because researchers had to concentrate vesicles from culture filtrates by several low- and high-speed centrifugation steps, and additional biochemical tools (Lee *et al*., 2009; reviews: Kulp and Kuehn, 2010; Manning and Kuehn, 2013; Berleman *et al*., 2014). In contrast, we avoid these harmful procedures, and hence, succeed to monitor the *in vivo* generation of vesicles of a *Streptomyc*es strain being also the first example among bacteria.

The vesicle-containing droplets of *S. lividans* contain many vesicles (∼10^11^ μl^−1^) that are like those from *S. coelicolor* M110 (Schrempf *et al*., 2011) extremely protein rich (1–2 μg μl^−1^). In contrast, researchers obtained only small amounts (30 μg) of proteins from OMVs that they gained from 1 l culture filtrate of *Xanthomonas campestris* (Sidhu *et al*., 2008). In addition, to specific vesicle proteins, culture filtrate-derived OMV-preparations from Gram-negative bacteria have varying quantities of proteins that usually reside in the cytoplasm, or in the inner membrane. It is still unclear, if these are true components or contaminants (review: Manning and Kuehn, 2013). Likewise, different cytoplasmic proteins are present in MV preparations obtained from culture filtrates of the Gram-positive *S. aureus*; these comprise ribosomal proteins, elongation factors, tRNA synthetases, subunits of DNA-dependent RNA polymerase, glyceraldehyde-3-phosphate-dehydrogenase and pyruvate kinase (Lee *et al*., 2009).

In contrast, the *Streptomyces* vesicle-containing exudates are devoid of these typical cytoplasmic proteins, and include under the same growing conditions reproducibly specific proteins, many of which are enzymes and binding proteins (Table 1). From these, 59% have either a predicted Sec (23%), or respectively, a Tat (35%) signal peptide. The other types (41%) lack this feature; these have predicted transmembrane region(s), or a lipid anchor, or none of these traits. None of the proteins carries a C-terminal motif for a sortase-dependent anchoring at the cell wall (Table 1).

The genomic information predicts for streptomycetes many (∼800) secreted proteins from which about 15% are deduced to be secreted in a Tat-dependent fashion. The engineered overexpression of genes encoding the Tat machinery resulted in a reduced secretion of Sec-dependent proteins (De Keersmaeker *et al*., 2006). We conclude that the transcription of genes encoding the recorded vesicle proteins (Table 1) with a Tat signal peptide (35%) is upregulated, and may correlate with locally enhanced levels of the dynamic Tat machinery when the formation of vesicles is initiated. The identified vesicle proteins that lack the above-outlined signatures (Table 1) likely belong to the growing class of proteins that depend on the so-called non-classical secretion (reviews: Bendtsen *et al*., 2005; Chagnot *et al*., 2013; Schrempf *et al*., 2011).

In contrast to Gram-negative bacteria, Gram-positive ones lack a periplasmic space and thus, a compartment in which the interaction of nascent secreted polypeptides with folding and processing factors occurs. A solution to overcome this deficit is ExPortal, which researchers have recently detected in the Gram-positive bacterium *Streptococcus pyogenes*. ExPortal is a focal microdomain with enhanced levels of anionic phospholipids that is required to cluster translocons of the general secretory pathway, and factors for the maturation of the nascent secreted polypeptides. Proteins that are necessary for membrane-associated steps of lipid II (see above) localize to foci coinciding with the anionic lipid microdomain. The data indicate an association of ExPortal and cell wall synthesis (Vega *et al*., 2013; Port *et al*., 2014). Interestingly, the *Streptomyces v*esicles emerge at local sites with enhanced levels of anionic phospholipids and lipids that correlate with the positioning of lipid II (see above; Fig. 4).

Peptidyl-prolyl-isomerases catalyse a high rate of *cis-trans* isomerization of peptidylproline that play possible roles in protein translation, folding, assembly and trafficking (Golbik *et al*., 2005). The membrane-anchored PrsA protein from *B. subtilis* belongs to this enzyme class, and determines the posttranslational folding and stability of exported penicillin-binding proteins that catalyse cross-linkages within peptidoglycan (Hyyryläinen *et al*., 2010). We assume that the identified peptidyl-prolyl-isomerase-type (D6ENL9) assists the folding of proteins during their sorting into the *S. lividans* vesicles. During this process, the predicted pyridine-nucleotide-disulphide oxidase (D6EHM2) may participate in the formation of disulfide bonds, a homologue with this function occurs within eukaryotes (Bardwell, 2002).

The application of vesicle containing *Streptomyces* droplets provokes clumping of *E. coli* cells (Fig. 6A and B) that kept their viability. Vesicles appear to fuse at sites along fungal hyphae of *A. proliferans* and *Verticillium dahliae*, and mediate severe cellular damages (Figs 7 and 8), which are probably due to the concerted action of several identified *S. lividans* proteins (Table 1), and the identified undecylprodigiosin. The peptidases (WP016326180 and D6EU9) can participate in the degradation of structural cell proteins including those fungi. The biocontrol fungus *Trichoderma harzianum* secretes an array of different peptidases that researchers have implicated to play important roles in the antagonistic repertoire (Suárez *et al*., 2007). The alkaline phosphatase type (D6EJ35) is a novel member of the metallophosphatase superfamily that we have recently characterized. As it diverges from other known phosphatase types, we named the protein MptS (metallophosphatase type from *Streptomyces*). Previously, we revealed that MptS supports the interaction among *S. lividans* spores with conidia of *A. proliferans* (Lamp *et al*., 2013). The MptS protein (D6EJ35) gained from vesicles is enzymatically active as it releases inorganic phosphate from an artificial model substrate. Hence, the MptS can support the harmful effects of vesicles on microbes.

Phospholipids within cellular membranes are hydrolysable by a range of enzymes (Katan, 2005; Santos-Beneit *et al*., 2009; Krishnamoorthy *et al*., 2014). The identified *S. lividans* phospholipase C-type (D6EFC0) belongs to a large superfamily phosphoinositide-specific phosholipases. The identified vesicle protein D6ENU1 belongs to glycerophosphoryl-diester-phosphodiesterases that hydrolyses deacylated glycerophosholipids. Many pathogenic microbes produce phospholipase(s) that disrupt the host membranes, and the resulting lipids act as secondary messengers, and hence, provoke alterations within the signal transduction cascade (review: Djordjevic, 2010). The enzymatic action of agmatinase results in putrescine, the precursor of the polyamines, spermidine and spermine, and is known to stabilize phospholipids, nucleic acids and proteins in all kingdoms of life (Burnat and Flores, 2014). The agmatinase (D6EXY3) likely impairs the cellular levels of polyamines of microbes treated with vesicles.

The level of H_2_O_2_ is fine tuned within organisms. Hence, the vesicular catalase (D6EG84) enhances the level O_2_ that can result in an altered level of harmful reactive oxygen species. In addition, H_2_O_2_ operates as a signalling molecule (Sewelam *et al*., 2014). Nucleotide signalling plays an important role in tissues, differentiation or following pathogen infection (Rooklin *et al*., 2012; Porowińska *et al*., 2014). Based on these findings, we conclude that the identified *S. lividans* nucleotidase/apyrase (D6EK57) has a similar capacity. The nucleotide-binding protein (D6EUN8) likely takes part in a predicted signalling cascade.

Following the application of droplet containing vesicles, undecylprodigiosin-derived red fluorescence appeared sometimes within the fungus as round elements, and later in a diffused fashion. Undecylprodigiosin belongs to the class of prodiginines. These metabolites have multiple cellular actions, i.e. uncoupling of the lysosomal vacuolar-type ATPase, DNA intercalation, inhibition of various steps within the cell cycle leading to its arrest, apoptosis and possible interference with signal-transduction pathways (review: Williamson *et al*., 2006; Meschke *et al*., 2012). Previously, we described that *S. lividans* produces prodiginines during co-cultivation with *V. dahliae* in the presence or absence of plant roots from *Arabidopsis thaliana*. In addition, we revealed that purified undecylprodigiosin is taken up by hyphae of *V. dahliae*, and impairs microsclerotia formation, possibly following alteration(s) of signal transduction pathways (Meschke *et al*., 2012). In addition, vesicles contain a range of proteins interfering with signal transduction and degradation of cellular components (Table 1); hence, the cellular damages are more severe (Figs 6–8).

Proteins that are unique for the *S. lividans* vesicles (Table 1) comprise the nucleotidase/apyrase (D6EK57), the alkaline phosphatase (D6EJ35), the pyridine-nucleotide-disulphide oxidase (D6EHM2), the ketol-acid-reductoisomerase (D6ERQ9) catalysing the biosynthesis of intermediates for branched-chain aminoacids (Brinkmann-Chen *et al*., 2013), three different binding proteins (D6EUN8, D6EXX3 and D6E187) and the esterase (D6EKU8). Earlier, we had characterized this esterase type (named FusH) to cleave off acetyl, thioacetyl or a formyl group from the steroid-like antibiotic fusidic acid or its derivatives (von der Haar *et al*., 1997).

Noticeably, several proteins (Table 1) within the *S. lividans* vesicles-containing exudates (i.e. catalase, glycerophosphoryl-diester-phosphodiesterase, phosphate-binding protein, peptidyl-prolyl cis-trans isomerase, agmatinase and the TerD protein) correspond to those encountered within the vesicles from *S. coelicolor* M110 (Schrempf *et al*., 2011), and others (peptidases and phospholipase) are divergent subtypes. In contrast, some protein types reside only in vesicles of *S. coelicolor* M110 (Schrempf *et al*., 2011), but are absent in those of *S. lividans*. The cargo variation may hint to adaptations to different ecological niches, and divergent inter-and intra-species communications and competitions.

Summarizing, we pioneered to monitor the *in vivo* generation of vesicles of a *Streptomyc*es strain being also the first example among bacteria. The vesicle-containing protuberances arise at sites containing defects within the peptidoglycan layer of *Streptomyces* substrate hyphae that correlate with enhanced levels of lipids and phospholipids, and their local arrangement is likely important for the extrusion of vesicles. The dominance of TAT-dependent proteins and those lacking a signal peptide suggests a selective sorting process. The proper folding of vesicle proteins will likely depend on the identified peptidyl-prolyl-isomerase. In order to deduce generalities, more future investigations are necessary.

We revealed for the first time that the *Streptomyces* vesicles are potent to deliver cargo including a secondary metabolite to microbes. We suspect that this type of delivery will also take place under environmental conditions, and has a range of applications including the combat of harmful fungi.

## Experimental procedures

### Strains and cultivation

Cultivation of *S. lividans 66* (in the text named *S. lividans*, Hopwood *et al*., 1985) on Petri dishes containing complete medium (R-S) was described earlier (Koebsch *et al*., 2009). Stock suspensions containing 2.5 × 10^9^ spores per ml 40% glycerol were stored at −20°C. The growth conditions and the preparations of conidia of *A. proliferans* and, respectively, *Verticillium dahlia* were described earlier (Siemieniewicz, *et al*., 2007,2007; Meschke and Schrempf, 2010). *Escherichia coli* DH5α and *B. subtilis* 168 were cultivated in Luria–Bertani medium (Koebsch *et al*., 2009).

### Chemicals, dyes and enzymes

Chemicals for SDS gel electrophoresis were from Serva, and Sepharyl S-300 was from GE Healthcare Life Sciences. Other chemicals were purchased from Sigma. Trypsin was from Roche. The dyes Nile red, NAO and Van-FL (BODIPY FL vancomycin) were purchased from Sigma, AA Bioquest and Invitrogen respectively.

### Microscopy under visual and fluorescence light and counting of particles

Portions of plates containing droplets (see Fig. 1A) were photographed (Canon Power Shot G2) using a macro lens (dioptre value of + 4). Samples of the droplets or aliquots (500 μl) of cultures from *S. lividans*, *E. coli*, *B. subtilis*, *A. proliferans* or *V. dahliae* [exposed to droplets (20 μl) or not] were analysed under visible light at various magnifications using a Zeiss Observer Z1 microscope. To test for the presence of undecylprodigiosin, samples were examined under reflected light using filter sets (for Texas Red: excitation: 562/40, beam splitter: 593 LP, emission: 624/40 from AHF Analysentechnik AG) attached to a Zeiss Observer Z1 microscope as described earlier (Meschke and Schrempf, 2010).

We performed LIVE/DEAD staining, and investigated the results by fluorescence microscopy using the previously reported dyes (Berney *et al*., 2007; Siemieniewicz and Schrempf, 2007).

We counted particles within many equally framed squared areas on photographs (Fig. 1C and D) taking the volume of the samples in an account. In addition, we compared the data with the numbers gained from electron micrographs (Fig 1E).

### Staining with Nile red, NAO and Van-FL (BODIPY FL vancomycin)

Samples of *S. lividans* were treated with Nile red, and NAO or Van-FL (BODIPY FL vancomycin), and analysed under the Zeiss Observer Z1 microscope for the presence of red, or respectively, green fluorescence as outlined previously (Daniel and Errington, 2003; Kumagai *et al*., 2005; Oliver *et al*., 2014). The following filter sets were used from AHF Analysentechnik AG: HQ 562/40, beam splitter Q: 593 LP, Q emission: HQ 624/40, and respectively, 470/40 beam splitter, Q 495 LP, emission: HQ 525/50 (Meschke and Schrempf, 2010).

### TEM

Samples of the droplets (5–10 μl) were pre-treated with glutaraldehyde (0.25%) for 15 min, and were placed onto carbon-covered Cu grids (300 mesh, Plano). After removal of the excess of liquid, neutralized phosphotungstic acid (3% W/V) was added for 1 min. Then, the grids were rinsed on drops of distilled water, air-dried and analysed with a Zeiss EM 902A, and pictures were imaged using a digitalized camera (Koebsch *et al*., 2009).

### Test for the presence of undecylprodigiosin

We enriched undecylprodigiosin as described earlier (Koebsch *et al*., 2009), and analysed the samples by LC/MS for the presence of undecylprodigiosin [ion m/z (394.4 M + H)^+^] as we have reported previously (Meschke *et al*., 2012).

### Analysis of proteins

A small portion (30 μl) from droplets was subjected to electrophoresis (12.5% SDS-PAGE). Each protein-containing band was treated with trypsin; the generated peptides were separated by HPLC (reversed phase C18 column), and inline subjected to an ESI-ion source mass spectrometer (Bruker HCT). The archived data were compared with a protein databank (Swissport) via Mascot software package. The identified protein sequences were analysed for their domains as well as the corresponding genes.
